# Factors associated with mood disorders and the efficacy of the targeted treatment of functional dyspepsia: A randomized clinical trial

**DOI:** 10.3389/fmed.2022.859661

**Published:** 2022-07-22

**Authors:** Qian Huang, Shaopeng Zheng, Ting Cai, Suxin Zhang, Qian Su, Fen Wang

**Affiliations:** ^1^Department of Gastroenterology, The Third Xiangya Hospital, Central South University, Changsha, China; ^2^Hunan Key Laboratory of Non Resolving Inflammation and Cancer, Changsha, China

**Keywords:** dyspepsia, psychotropic medications, PPIs, H2RAs, efficacy

## Abstract

**Background:**

Patients with functional dyspepsia (FD) are often accompanied by mood disorders (MDs). This study aimed to identify factors associated with MDs in patients with FD and evaluate the efficacy of targeted treatment plans.

**Methods:**

Relevant scales were used to assess MDs. Patients with FD having MDs and acid reflux were treated with flupentixol and melitracen (FM) and acid-suppressive therapy (AST) (histamine-2 receptor antagonists (H2RAs) (group A) or proton pump inhibitors (PPIs) (group B)), and those without acid reflux (group C) did not receive AST. Patients with FD without MDs were randomly administered H2RAs (group D) or PPIs (group E). The primary endpoints were factors associated with MDs and improvement in gastrointestinal (GI) symptoms and MDs in patients with FD.

**Results:**

A total of 362 patients with FD were enrolled in this study. Patients with FD having high GI score and low education were found prone to MDs. At week 2, the remission rate of overall GI symptoms and depression was significantly higher in group B than that in groups A and C [GI: 72.72% (32/44) vs. 47.73% (21/44) and 72.72% (32/44) vs. 38.94% (44/113), all *P* < 0.05; depression: 72.22% (26/36) vs. 41.67% (15/36) and 72.22% (26/36) vs. 41.57% (37/89), all *P* < 0.05]. Furthermore, the remission rate of overall GI symptoms was significantly higher in group E than that in group D [60.29% (41/68) vs. 42.65% (29/68), *P* < 0.05]. At week 8, similar efficacies and adverse reactions were observed in these groups.

**Conclusion:**

The risk factors for MDs were high GI scores and low literacy rates. Thus, targeted treatment (FM+PPIs for patients with MDs; PPIs for patients without MDs) can improve the efficacy of patients with FD.

**Clinical trial registration:**

www.chictr.org.cn, identifier ChiCTR2100053126.

## Introduction

Functional dyspepsia (FD) is a common gastrointestinal (GI) disorder, with a 5–40% prevalence based on differences in definition criteria and geographic location (1). The main clinical symptoms of FD are postprandial fullness, early satiety, epigastric pain and epigastric burning, which are accompanied by belching and acid regurgitation. FD is divided into three subtypes according to its symptoms: postprandial distress syndrome (PDS) (postprandial fullness and early satiety), epigastric pain syndrome (EPS) (epigastric pain and burning) and overlapping FD (one or two symptoms of both PDS and EPS). Acid suppressive therapy (AST) and GI motility drugs are used to treat EPS and PDS, respectively.

The ineffective treatment of FD places an undue economic and psychological burden on affected patients. Although psychotropic drugs can effectively improve GI symptoms in patients with FD (2–4), their effectiveness is limited to patients with FD having mood disorders (MDs) (5). This could be attributed to the gastrointestinal manifestations caused by the somatisation of mental illness in some patients with FD (6). Thus, targeted FD treatment based on the presence of MD is vital.

However, the therapeutic efficacy of different psychotropic medications in patients with FD varies (4, 7, 8). It has been reported that the combination of flupentixol and melitracen (FM) has both anxiolytic and antidepressant properties (9), which can be used for the treatment of refractory FD. Additionally, it is also efficient for other patients with MDs, such as patients with gastroesophageal reflux (10), chronic physical diseases (11) and dizziness (12). Moreover, the effectiveness of proton pump inhibitors (PPIs) and histamine-2 receptor antagonists (H2RAs) for FD is controversial (13, 14). The adverse effects of PPIs include bone fractures, *Clostridium difficile* infections and pneumonia (15–17) while those of H2RAs induce delirium and multiple cerebral infarctions (18, 19).

This clinical study aimed to identify more effective treatment strategies, including psychotropic drugs and AST, for patients with FD with and without MDs and thereby improving symptoms and reducing psychological burdens.

## Research subjects and methods

### Subjects

This study included patients with FD receiving treatment in the Department of Gastroenterology of Third Xiangya Hospital of Central South University from January 2019 to November 2020. The inclusion criteria were as follows: the presence of epigastric pain, epigastric burning, postprandial fullness or early satiety for at least 6 months (from the time of onset until diagnosis); negative results on gastroscopy, abdominal ultrasound, serological tests and ^14^C urea breath test (20). The exclusion criteria included alarm symptoms, such as anemia or gastrointestinal bleeding; symptoms suggestive of an eating disorder or gastroparesis, including recurrent vomiting or unexplained weight loss of more than 10% of body weight in the past 3 months (21); prior history of major gastrointestinal surgery or presence of a major medical illness; history of organic gastrointestinal disease (e.g., peptic ulcer, reflux esophagitis, erosive gastritis and pancreatitis); diagnosed with anxiety or depression and currently undergoing antidepressant or antipsychotic therapy; use of AST or psychotropic drugs within 2 weeks prior to enrolment; concurrent use of monoamine oxidase inhibitors; concomitant coronary heart disease or heart block; multiple chronic diseases; history of drug or alcohol abuse (22); allergies to research medication; untreated angle-closure glaucoma; pregnancy or breastfeeding; inability to communicate effectively. All patients provided informed consent before their inclusion in the study.

### Study design

This was a single-center, positive-control, randomized, open study in patients with FD.

Based on the principle of complete randomization, a statistician used the random number generator in the SPSS software version 25.0 to generate two randomization lists, each having 200 random numbers. Random grouping was achieved at a ratio of 1:1. Subsequently, 200 opaque envelopes were sequentially numbered (001–200) and the corresponding randomized grouping result (group A or B) was packed, according to one randomization list. All envelopes were placed in a carton labeled “FD with MDs and Acid Reflux Symptoms.” Similarly, another randomized grouping result (group D or E) was packed into the other 200 opaque envelopes and placed in a carton labeled “FD without MDs.” The randomization process was performed independently by a statistician, and study interveners were blinded to the results of the treatment.

**Figure 1:**
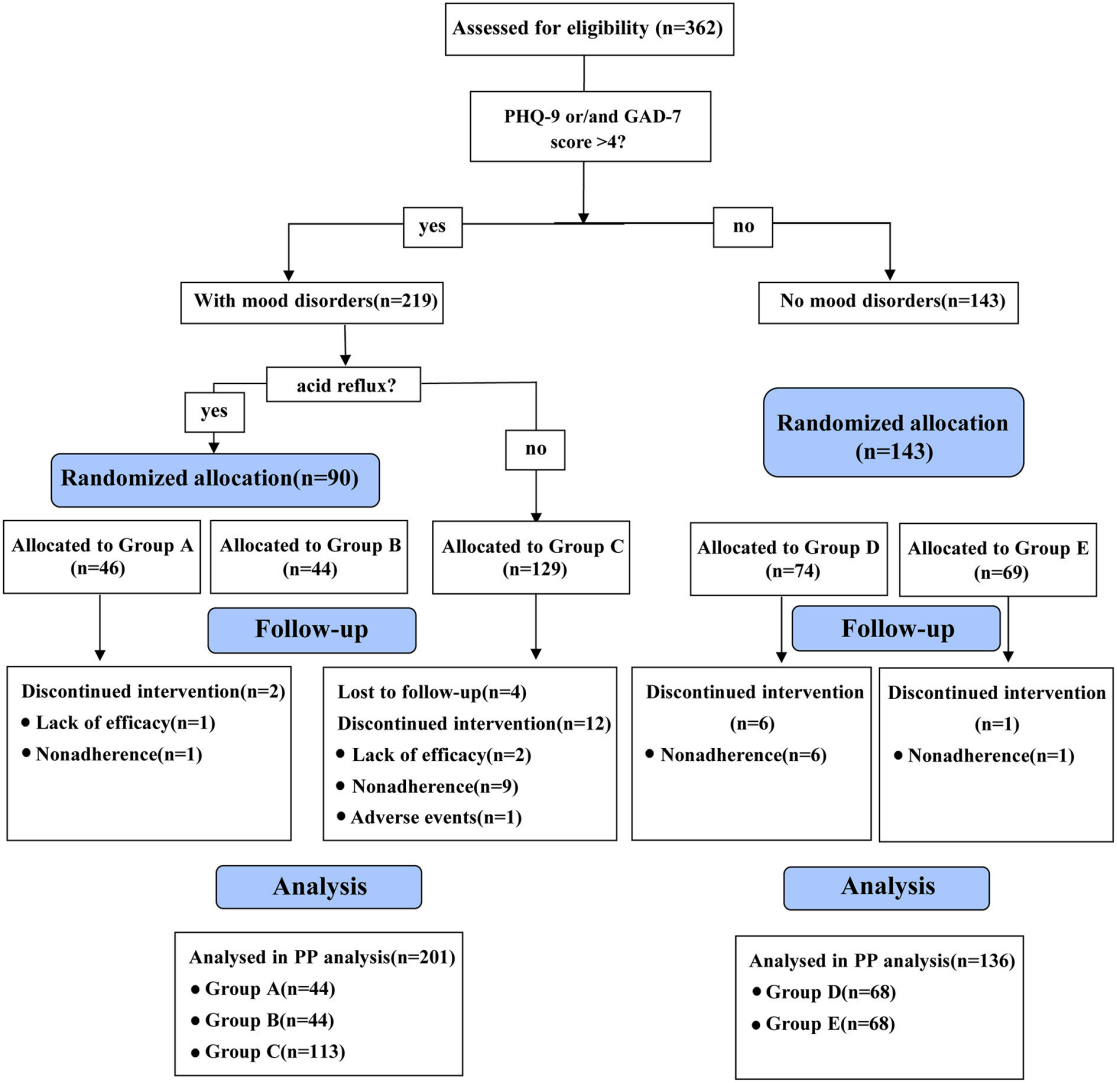
Flowchart of the recruitment and assignment of patients with functional dyspepsia. PHQ-9, patient health questionnaire-9; GAD-7, generalized anxiety disorder-7; Group A: flupentixol and melitracen + nizatidine; Group B: flupentixol and melitracen + rabeprazole; Group C: flupentixol and melitracen. PP, per protocol.

Demographic and clinical data were obtained using a self-administered questionnaire (Supplementary Table 1). Meanwhile, the mood state of patients with FD was evaluated using the Patient Health Questionnaire-9 (PHQ-9) and Generalized Anxiety Disorder-7 (GAD-7) screening questionnaires (Supplementary Tables 2A,B) under the guidance of a psychiatric professional. The patients with PHQ-9 or/and GAD-7 scores> 4 were considered to have MDs. After selecting patients with FD that met the above conditions, the research intervener opened the corresponding envelopes following the order of visits and delivered the corresponding prescription. In this study, all patients with FD having MDs were treated with psychotropic medications. The treatment allocation is shown in Figure 1. Additionally, all patients simultaneously received probiotics and digestive enzymes for 8 weeks. Treatment efficacy for GI symptoms and MDs were evaluated at weeks 2, 4 and 8.

This study was conducted in accordance with the Declaration of Helsinki and the Good Clinical Practice of the International Conference on Harmonization and approved by the Ethics Committee of The Third Xiangya Hospital of Central South University (ChiCTR2100053126).

### Drug therapies

The main medications used in this study were FM (H. Lundbeck & Co. A/S) (10.5 mg twice daily), nizatidine (Weite Pharmaceutical Co., Ltd., Hunan) (150 mg twice daily) and rabeprazole (Zhuhai Rundu Pharmaceutical Co., Ltd.) (10 mg twice daily).

Complementary drugs included bifid triple viable (Shanghai Shangyaoxinyi Pharmaceutical Co., Ltd.) (420 mg thrice daily), Oryz-Aspergillus enzyme and pancreatin tablets (Nordmark Arzneimittel GmbH & Co. KG) (488 mg thrice daily).

### GI symptom and anxiety/depression scores

The following GI symptoms were assessed: postprandial fullness, early satiety, epigastric pain and burning, epigastric distention, nausea, belching, acid reflux, chest pain and burning, vomiting and bad breath. Scoring was done according to the number of symptoms and impact on daily life: 0 (none), 1 (a few symptoms, no impact), 2 (some symptoms, mild impact), 3 (several symptoms, significant impact). Depression was assessed using the PHQ-9 questionnaire (23) according to the following score: 0–4 points (none), 5–9 points (mild), 10–14 points (moderate), 15–19 points (moderate to severe), 20–27 points (severe). Anxiety was evaluated using the GAD-7 questionnaire according to the following score: 0–4 points (none), 5–9 points (mild), 10–13 points (moderate), 14–18 points (moderate to severe), 19–21 points (severe).

### Endpoints

The primary endpoints were risk factors for MDs in patients with FD and the remission rate of GI symptoms and MDs. Therapeutic efficacy was classified according to the Clinical Global Impressions Efficacy Index [(pre-treatment score – post-treatment score)/pre-treatment score] as ineffective (0–24.9%), moderately effective (25.0–49.9%), effective (50.0–74.9%) or very effective (75.0–100%). The remission rate was defined as the proportion of patients with a complete (very effective) or partial (effective) treatment response.

The secondary endpoint was the rate of adverse events, which were recorded throughout the treatment period.

### Statistical analyses

Statistical analysis was performed using the SPSS software version 25.0. On the basis of clinical experience and the feasibility of this study, the sample size of groups A, B, D and E was set in the range of 40–100 cases, and the sample size of group C was not <40 cases, with a total of 300–400 participants. Among them, the sample size ratio for groups A and B and groups D and E was 1:1.

In the primary endpoint, factors associated with MDs were evaluated using logistic regression analysis. The Chi-square test or Fisher's exact test was used to compare and analyse the remission rate of GI symptoms and MDs and also the adverse reaction rate between treatment groups. Bonferroni correction was used for the adjustment of several statistical group comparisons. Additionally, intergroup remission rates at each time point were compared using generalized estimating equations. Baseline characteristics were analyzed using independent-samples *t*-test, Mann–Whitney U test, Kruskal–Wallis test, chi-square test or Fisher's exact test.

## Results

### Recruitment and grouping of patients with FD

The flowchart for the recruitment and assignment of patients with FD is detailed in Figure 1. Patients who were lost to follow-up (*n* = 4), were non-adhering (*n* = 17) and changed their medications due to poor efficacy (*n* = 3) or side effects (*n* = 1) were excluded. Among them, non-adherence was the main reason for the discontinuation of the trial. Finally, 337 patients completed the study, of whom 44, 44, 113, 68 and 68 were in groups A, B, C, D and E, respectively.

### Analysis of risk factors for MDs in patients with FD

#### Univariate analysis

Univariate analysis showed that MDs in patients with FD were correlated with high GI symptom score, body mass index, educational level, early satiety and epigastric burning (Table 1). Different types of MDs (depression, anxiety or comorbid depression and anxiety) were associated with gender (*P* = 0.030), GI symptom score (*P* = 0.002), number of previous treatments (*P* = 0.041), FD subtype (*P* = 0.018), early satiety (*P* = 0.024), epigastric distention (*P* = 0.048) and nausea (*P* = 0.043).

**Table 1:** Univariate analysis of risk factors for mood disorders in patients with functional dyspepsia.

**Variables**	**FD without MDs (*****n*** = **143)**	**FD with MDs** **(*****n*** = **219)**	***P*** **-value**
**Sex (*n*)**			0.119
Male	68	86	
Female	75	133	
**Age, years (median, Q1-Q3)**	46.00 (35.00–55.00)	45.00 (32.00–54.00)	0.237
**BMI, kg/m^2^ (median, Q1-Q3)**	22.48 (19.84–24.51)	21.45 (19.52–23.56)	0.021
**GI symptom score (median, Q1-Q3)**	6.00 (4.00–9.00)	8.00 (6.00–11.00)	<0.001
**Marital status (*n*)**			0.112
Others	15	36	
Married	128	183	
**Duration of disease onset, years (*n*)**			0.349
0.5-0.9	52	66	
1.0-4.9	52	100	
5.0-9.9	20	25	
≥10	19	28	
**Number of previous treatments (*n*)**			0.901
<3	78	118	
≥3	65	101	
**Educational Level (*n*)**			0.027
Primary	7	29	
Secondary	67	101	
Higher	69	89	
**Employed (*n*)**			0.342
No	39	70	
Yes	104	149	
**FD subtype (*n*)**			0.102
PDS	41	50	
EPS	30	34	
Overlapping subtype^†^	72	135	
**Cardinal symptom (*n*)**			
Postprandial fullness	101	170	0.134
Early satiety	50	101	0.035
Epigastric pain	87	143	0.389
Epigastric burning	35	88	0.002
Epigastric distention	70	130	0.052
Nausea	39	78	0.097
Belching	75	125	0.386
Acid reflux	54	90	0.526

*FD, functional dyspepsia; MDs: mood disorders; Q, quartile; BMI, body mass index; GI, gastrointestinal; PDS, postprandial distress syndrome; EPS, epigastric pain syndrome.^†^One or two symptoms of both PDS and EPS*.

#### Multivariate analysis

Multivariate analysis indicated that the risk factors for MDs in this study were high GI symptom scores [odds ratio (OR):1.13, 95% confidence interval (CI): 1.05–1.21] and low education (OR: 0.67, 95% CI: 0.47–0.95) (Table 2). Moreover, male patients (OR: 3.80, 95% CI: 1.48–9.75) and patients with PDS (OR: 2.30, 95% CI: 1.00–5.29) were more likely to be depressed. Patients whose number of previous treatments was <3 were more prone to anxiety (OR: 3.50, 95% CI: 1.38–8.89). Furthermore, patients with overlapping FD (OR: 2.30, 95% CI: 1.00–5.29) tended to have comorbid anxiety and depression.

**Table 2:** Multivariate analysis of risk factors for mood disorders in patients with functional dyspepsia.

**Variables**	β	**SE**	**Wald** χ^2^	***P*** **-value**	**OR**	**95% CI**
BMI, kg/m^2^	−0.06	0.04	2.89	0.089	0.94	0.87–1.01
GI symptom score	0.12	0.04	11.29	0.001	1.13	1.05–1.21
Educational level	−0.40	0.18	5.03	0.025	0.67	0.47–0.95
Early satiety	0.13	0.26	0.26	0.609	1.14	0.69–1.91
Epigastric burning	0.41	0.27	2.34	0.126	1.51	0.89–2.55

*β, regression coefficient; SE, standard error; OR, odds ratio; CI, confidence interval; BMI, body mass index; GI, gastrointestinal*.

### Baseline characteristics, treatment efficacy and adverse events in patients with FD with MDs

#### Baseline characteristics

No significant difference was observed in the baseline data between groups A, B and C, except for the acid reflux score (Supplementary Table 3).

#### Therapeutic efficacy of GI symptoms

**Figure 2:**
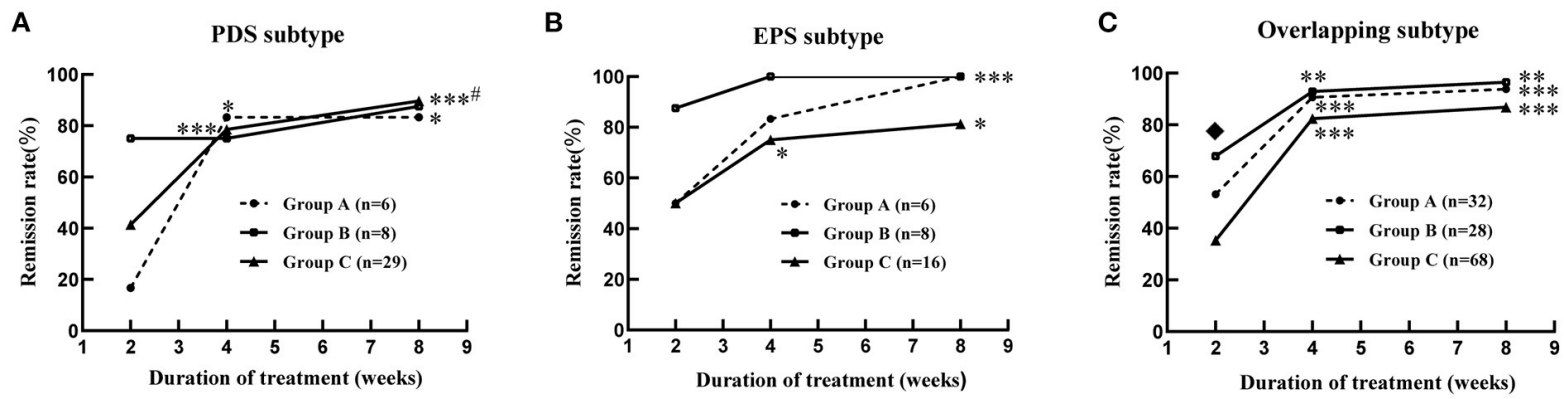
Improvement of global gastrointestinal symptoms in the patients with functional dyspepsia of different subtypes combined with mood disorders at 2, 4 and 8 weeks of treatment. **(A)** PDS subtype; **(B)** EPS subtype; **(C)** overlapping subtype. Group A: flupentixol and melitracen + nizatidine; Group B: flupentixol and melitracen + rabeprazole; Group C: flupentixol and melitracen. PDS, postprandial distress syndrome; EPS, epigastric pain syndrome; Overlapping subtype: one or two symptoms of both PDS and EPS. *P < 0.05, **P < 0.01, ***P < 0.001: vs. 2 weeks of treatment in the same group. ^#^*P* < 0.05: vs. 4 weeks of treatment in the same group. ^♦^*P* < 0.05: group B (19/28) vs. group C (24/68) at 2 weeks of treatment.

By comparing remission rates between groups A, B, and C, we found that at week 2 of treatment, therapeutic efficacy was significantly higher in group B compared to groups A and C [72.72% (32/44) vs. 47.73% (21/44) and 72.72% (32/44) vs. 38.94% (44/113), respectively, all *P* < 0.05, Table 3], especially in patients with overlapping FD [67.86% (19/28) and 35.29% (24/68) in groups B and C, respectively, *P* < 0.05, Figure 2C]. At 4 and 8 weeks, no significant difference was observed in the remission rate of overall GI symptoms among the three groups (Table 3). The remission rates of major GI symptoms, such as postprandial discomfort, early satiety, epigastric pain and epigastric burning, epigastric distention, nausea and belching, were also similar between groups A, B and C at week 8 (Supplementary Figure 1). Additionally, group C showed the best relief in overall GI symptoms at week 8 of treatment (Table 3 and Figure 2A), whereas groups A and B showed a good response at week 4 weeks, which was similar to the week 8 results (Table 3 and Figures 2A–C). Finally, the gender differences in overall GI symptoms remission at different treatment times were analyzed in the three treatment groups respectively considering the greater number of female patients with MD. Table 4 shows that the overall GI symptom remission rate of female patients with FD in group C was significantly higher than that of male patients at week 2 [48.48% (32/66) vs. 25.53% (12/47), *P* = 0.014], week 4 [86.36% (57/66) vs. 70.21% (33/47), *P* = 0.036] and week 8 [93.94% (62/66) vs. 76.60% (36/47), *P* = 0.007]. However, in groups A and B, remission rates were similar for male and female patients.

**Table 3:** The remission rate of global gastrointestinal symptoms in patients with functional dyspepsia with mood disorders (*n* [%]).

**Treatment duration** **(weeks)**	**Group A** **(*****n*** = **44)**	**Group B** **(*****n*** = **44)**	**Group C** **(*****n*** = **113)**
2	21 (47.73)	32 (72.72) ^‡, §^	44 (38.94)
4	39 (88.64)***	40 (90.91)**	90 (79.65)***
8	41 (93.18)***	42 (95.45)**	98 (86.73) ^***#^

Group A: flupentixol and melitracen + nizatidine; Group B: flupentixol and melitracen + rabeprazole; Group C: flupentixol and melitracen. ^‡^P < 0.05: vs. group A at 2 weeks of treatment, ^§^P < 0.05: vs. group C at 2 weeks of treatment. ^**^P < 0.01, ^***^P < 0.001: vs. 2 weeks of treatment in the same group. ^#^P < 0.05: vs. 4 weeks of treatment in the same group.

**Table 4:** Gender differences in overall gastrointestinal symptom improvement in patients with FD with mood disorders.

**Treatment** **duration (weeks)**	**Group** **A**^1^	**Group** **A**^2^	***P***	**Group** **B**^1^	**Group** **B**^2^	***P***	**Group** **C**^1^	**Group** **C**^2^	***P***
2	35.29 (6/17)	55.56 (15/27)	0.19	68.42 (13/19)	76.00 (19/25)	0.58	25.53 (12/47)	48.48 (32/66)	0.014
4	94.12 (16/17)	85.19 (23/27)	0.35	94.74 (18/19)	88.00 (22/25)	0.62	70.21 (33/47)	86.36 (57/66)	0.036
8	94.12 (16/17)	92.59 (25/27)	1.00	100.00 (19/19)	92.00 (23/25)	0.50	76.60 (36/47)	93.94 (62/66)	0.007

*Group A: flupentixol and melitracen + nizatidine; Group B: flupentixol and melitracen + rabeprazole; Group C: flupentixol and melitracen. ^1^Remission rates of overall gastrointestinal symptoms in male patients with FD; ^2^Remission rates of overall gastrointestinal symptoms in female patients with FD. Remission rate (%)*.

#### Remission rate of MDs

**Figure 3:**
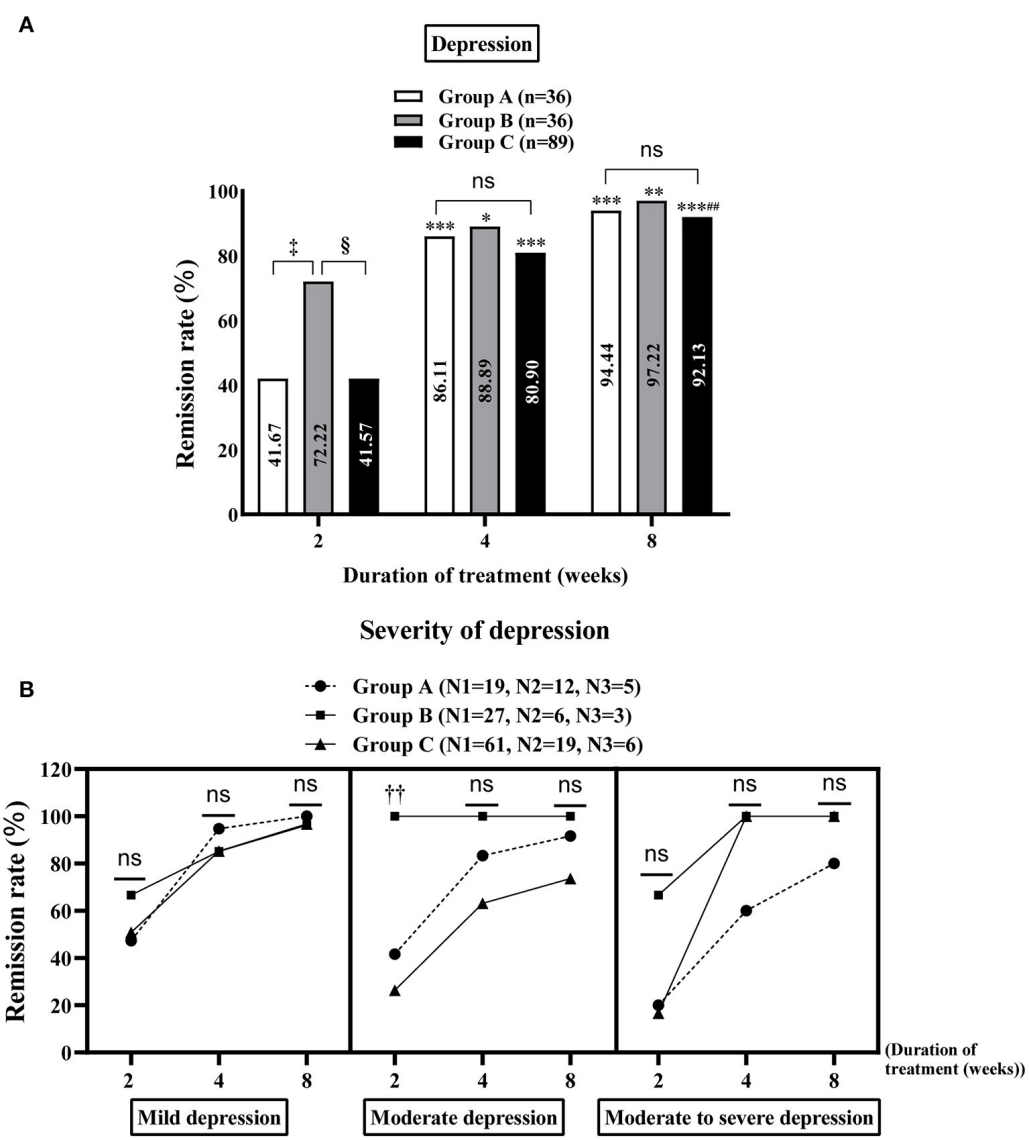
Improvement of depression in the study population. Comparison of the remission rates of general depression **(A)** and different degrees of depression **(B)** in groups A, B and C at 2, 4 and 8 weeks of treatment. Group A: flupentixol and melitracen + nizatidine; Group B: flupentixol and melitracen + rabeprazole; Group C: flupentixol and melitracen. N1, N2 and N3 correspond to the number of patients with mild, moderate and moderate/severe depression, respectively. *P < 0.05, **P < 0.01, ***P < 0.001: vs. 2 weeks of treatment in the same group. ^##^*P* < 0.01: vs. 4 weeks of treatment in the same group; ^‡^*P* < 0.05: vs. group A at 2 weeks of treatment, ^§^*P* < 0.05: vs. group C at 2 weeks of treatment. ^††^*P* < 0.05: group B [100% (6/6)] vs. group C [26.32% (5/19)] in treating moderate depression at week 2. Ns: no significance between groups A, B and C.

At week 2 of treatment, the remission rate of depression was notably higher in group B than in groups A and C [72.22% (26/36) vs. 41.67% (15/36) and 72.22% (26/36) vs. 41.57% (37/89), all *P* < 0.05, Figure 3A], especially moderate depression [100% (6/6) and 26.32% (5/19) in groups B and C, respectively, *P* < 0.05, Figure 3B]. On comparing groups A, B and C at week 8 of treatment, no significant difference was observed in the remission rate of MDs (anxiety and/or depression) [95.45% (42/44), 97.73% (43/44) and 94.69% (107/113), respectively, Supplementary Table 4], depression [94.44% (34/36), 97.22% (35/36) and 92.13% (82/89), respectively, Figure 3A] and anxiety [94.59% (35/37), 96.88% (31/32) and 93.75% (75/80), respectively, Supplementary Figure 2]. The results of the intra-group comparison showed that at week 4 of treatment, group B showed significant improvement in the MDs [week 4 vs. week 2: 93.18% (41/44) vs. 68.18% (30/44), *P* < 0.01, Supplementary Table 4], especially depression [week 4 vs. week 2: 88.89% (32/36) vs. 72.22% (26/36), *P* < 0.05, Figure 3A] and anxiety [week 4 vs. week 2: 90.63% (29/32) vs. 65.63% (21/32), *P* < 0.01, Supplementary Figure 2] in patients with FD, with similar effects observed at week 8. However, groups A [week 8 vs. week 4: 95.45% (42/44) vs. 86.36% (38/44), *P* < 0.05, Supplementary Table 4] and C [week 8 vs. week 4: 94.69% (107/113) vs. 81.42% (92/113), *P* < 0.01, Supplementary Table 4] required 8 weeks of treatment to achieve better results. Additionally, gender differences in each treatment group in alleviating MDs in patients with FD were explored. The results showed that although there was no significant difference in the relief of MDs and anxiety in groups A, B and C between male and female patients with FD (Supplementary Tables 5A,C), the improvement of depression in female patients with FD in group C was significantly better than that in male patients at week 2 of treatment [53.06% (26/49) vs. 27.50% (11/40), *P* = 0.015, Supplementary Table 5B).

#### Adverse events

The rate of adverse events in groups A, B and C was 15.91% (7/44), 13.64% (6/44), and 10.62% (12/113), respectively, but no significant differences were found (*P* = 0.64). The most common adverse events were thirst and constipation, and the least common events were skin pruritus and increased defecation.

### Baseline characteristics, treatment efficacy and adverse events rate in patients with FD without MDs

**Figure 4:**
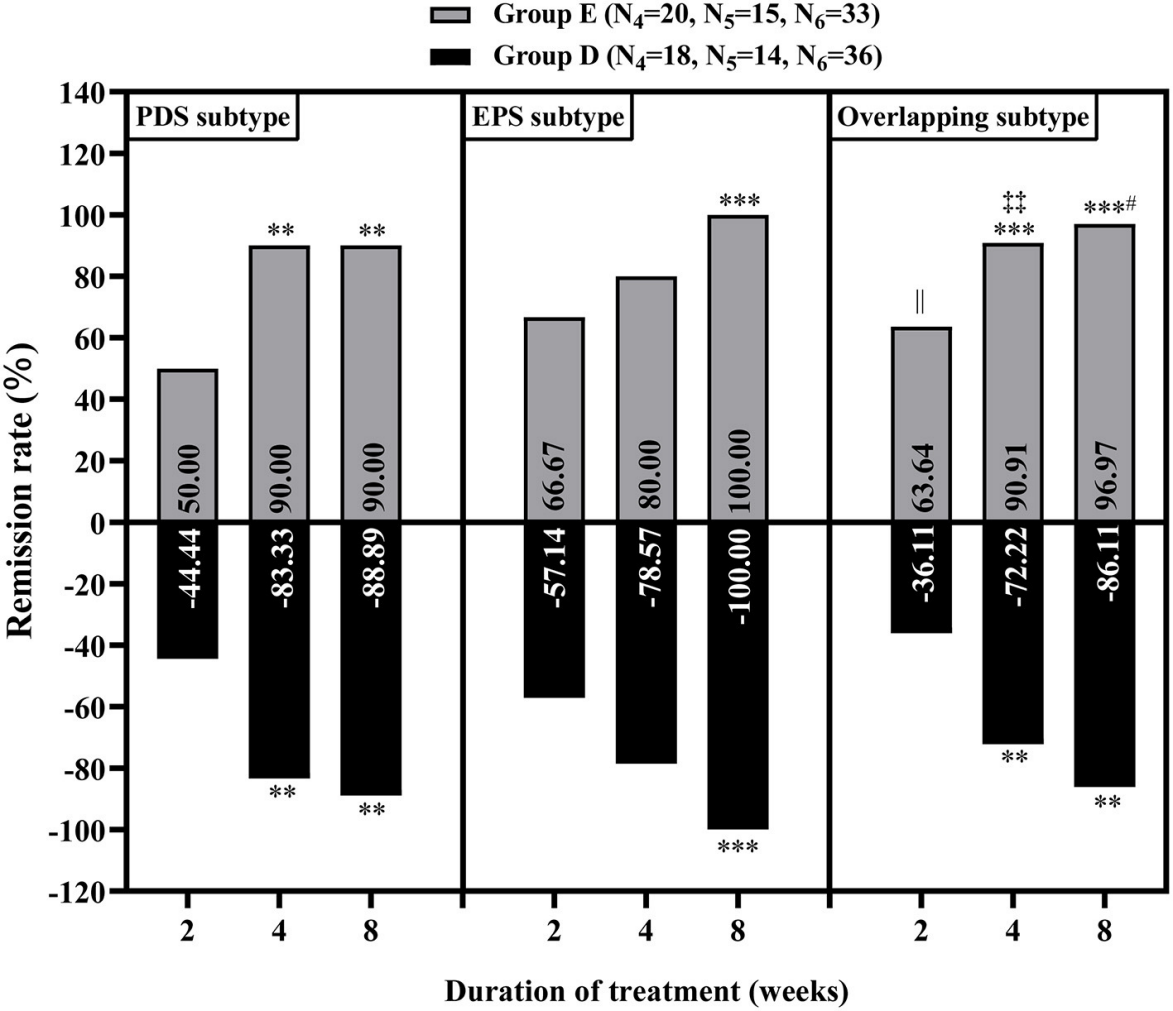
Relief of overall gastrointestinal symptoms in the patients with functional dyspepsia of different subtypes without mood disorders at 2, 4 and 8 weeks of treatment. Group D: nizatidine, Group E: rabeprazole. N4, N5 and N6 correspond to the number of patients with postprandial distress syndrome (PDS), epigastric pain syndrome (EPS) and overlapping subtype (one or two symptoms of PDS and EPS), respectively. **P < 0.01, ***P < 0.001: vs. 2 weeks of treatment in the same group; ^#^*P* < 0.05: vs. 4 weeks of treatment in the same group; ^||^*P* < 0.05: group E vs. group D at 2 weeks of treatment; ^‡‡^*P* < 0.05: group E vs. group D at 4 weeks of treatment.

**Figure 5:**
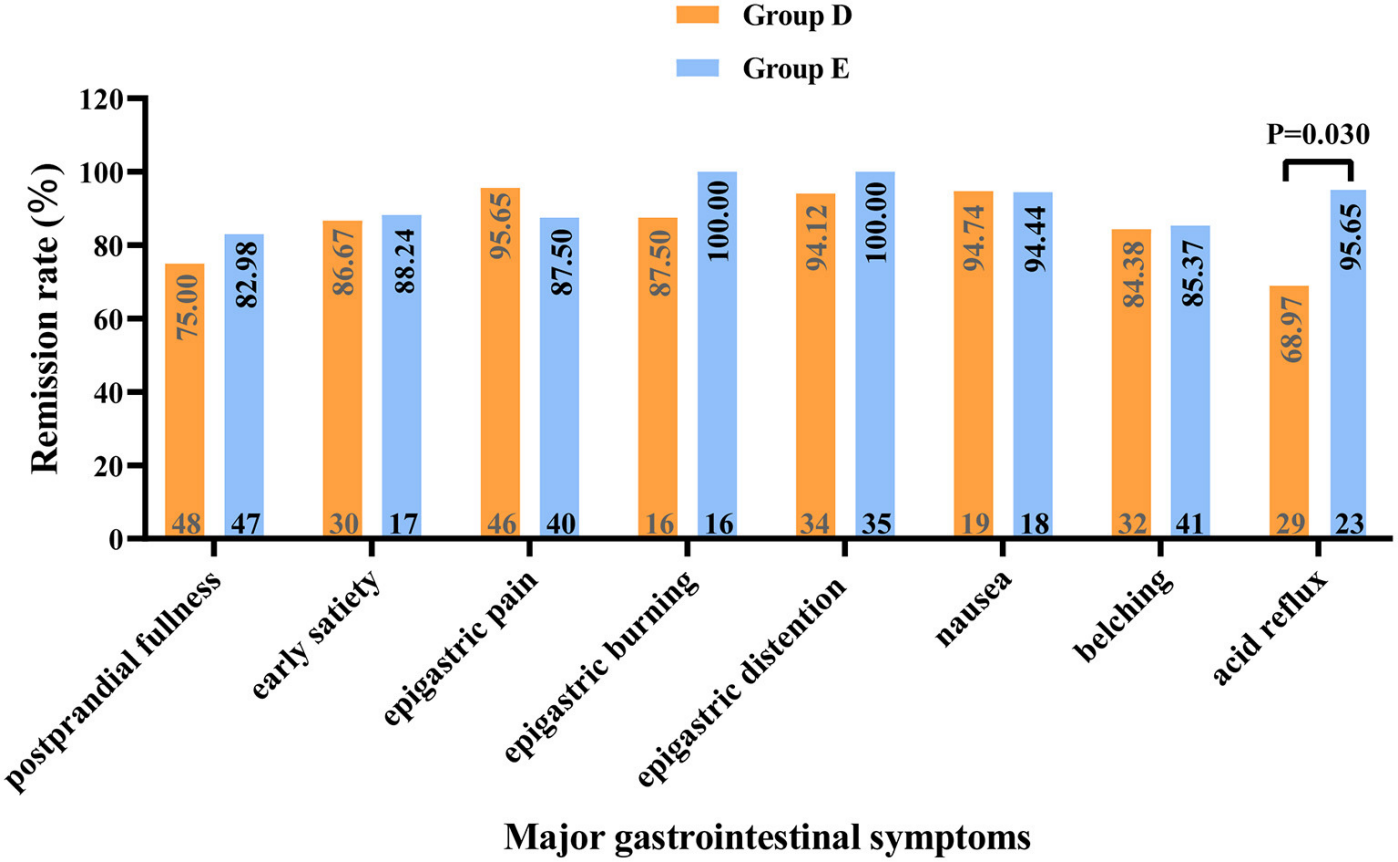
Relief of major gastrointestinal symptoms in patients with functional dyspepsia without mood disorders at 8 weeks of treatment. The numbers at the top and bottom of each column correspond to the remission rate and the number of patients with the respective symptom (before treatment). Group D: nizatidine; Group E: rabeprazole.

There was no significant difference in baseline data between the groups without MDs (*P* > 0.05, Supplementary Table 6). Through the comparison between groups D and E, we found that at week 2 of treatment, group E showed better improvement in the overall GI symptoms of patients with FD than group D [60.29% (41/68) vs. 42.65% (29/68), *P* < 0.05, Table 5]. Meanwhile, among patients with FD showing overlapping subtypes, the therapeutic efficacy of group E on overall GI symptoms was also significantly higher than that of group D at weeks 2 and 4 of treatment [63.64% (21/33) vs. 36.11% (13/36) and 90.91% (30/33) vs. 72.22% (26/36), respectively, all *P* < 0.05, Figure 4]. However, at week 8 of treatment, the remission rate of overall GI symptoms was similar between the groups D and E, irrespective of FD type (Figure 4). Regarding the main GI symptoms of FD, the efficacy of antiacid reflux was higher in group E than in group D [95.65% (22/23) vs. 68.97% (20/29), Figure 5]. Additionally, the results of the intra-group comparison showed that groups D and E usually achieved better efficacy at week 8 of treatment (Table 4 and Figure 4). The rate of adverse events was similar between groups D and E [10.29% (7/68) and 2.94% (2/68), respectively, *P* = 0.17]. The most common adverse reactions were dry mouth, constipation and loose stool.

**Table 5:** The remission rate of overall gastrointestinal symptoms in patients with functional dyspepsia without mood disorders (n [%]).

**Treatment duration (weeks)**	**Group D** **(*****n*** = **68)**	**Group E** **(*****n*** = **68)**
2	29 (42.65)	41 (60.29)^||^
4	52 (76.47)***	60 (88.24)***
8	61 (89.71)^***##^	65 (95.59)^***#^

Group D: nizatidine; Group E: rabeprazole. ^||^P < 0.05: vs. group D at 2 weeks of treatment; ^***^P < 0.001: vs. 2 weeks of treatment in the same group; ^#^P < 0.05, ^##^P < 0.01: vs. 4 weeks of treatment in the same group.

## Discussion

The risk factors for MDs in patients with FD included high GI symptom scores and low literacy levels. Patients with FD are often accompanied by low-grade intestinal inflammation (24), which can induce depression and anxiety by increasing the number of circulating gut-homing T cells and the secretion of tumor necrosis factor-α, interleukin-1β and other immune factors secreted by the peripheral blood mononuclear cells (25, 26). Moreover, a low level of education was found to predispose patients to MDs, which is consistent with previous studies (27, 28). This could be related to the fact that patients with lower education levels tend to have a less healthy lifestyle and less awareness of depression (29). Notably, depression can also affect the educational achievement of children and adolescents (30). Furthermore, the male gender and PDS increased the risk of depression in the current study. The former could be related to the presence of a single nucleotide polymorphism (SNP). Powers et al. found that among males, rs6602398, an SNP located at the IL2RA chromosome position 10p15.1, was significantly associated with mood dysregulation in a genome-wide association study [β(SE) = 14.9 (2.6); *P* = 1.1 × 10^−8^]. Subsequent logistic regression analyses revealed that this SNP was significantly associated with depression [Exp(B) = 2.67, *P* < 0.001]. However, at a cut-off level of 5x10^−8^, no SNPs were found to be significantly associated with emotional dysregulation in women (31). Hence, the increased risk of depression among women reported previously (32, 33) could be attributed to increased focus on the psychological state of females relative to males (34). Furthermore, PDS increases the risk of depression, which is consistent with a previous study (35), and could be due to GI hypersensitivity (36, 37). In contrast, other studies found that impaired gastric accommodation, which was related to anxiety (38), was more prevalent in PDS than in EPS (39). Even, the FD subtype was not associated with anxiety or depression (40). Therefore, the relationship between FD subtypes and these MDs warrants further investigation. In summary, psychotherapeutic support should be considered as a potential treatment option for patients with PDS, severe GI symptoms and low education levels.

Notably, a small percentage of this study's subjects were recruited during the coronavirus disease 2019 (COVID-19) pandemic. Studies have confirmed that the COVID-19 pandemic and lockdown restrictions could have significantly increased the prevalence of FD or worsened existing gastrointestinal symptoms. Moreover, anxiety was significantly associated with the increased prevalence of FD and worsening symptoms (41, 42). This suggests that the COVID-19 pandemic or lockdown restrictions could have indirectly exacerbated MDs in patients with FD.

In this study, at week 8 of treatment, the antidepressant and antianxiety agents FM not only significantly improved patients' anxiety and depression, but also had an 86.73% remission rate of gastrointestinal symptoms and a low rate of adverse reactions. Therefore, FM can be considered an effective and safe drug for patients with FD. This result could be explained through the pharmacological mechanism of FM. Flupenthixol is a typical antipsychotic while melitracen is a bipolar thymoleptic with activating properties (9, 43). The drug combination improves dopamine, norepinephrine and serotonin levels in the brain and has a synergistic effect by blocking postsynaptic dopamine receptors, along with a few extrapyramidal and anticholinergic effects (9, 11). Additionally, this study confirmed that FM significantly improved GI symptoms in female patients with FD than in male patients within 8 weeks of treatment, suggesting that the efficacy of FM on FD could be affected by gender. Similarly, Yin et al. indicated that gender was one of the independent predictors of acupuncture treatment response and improvement in Nepean Dyspepsia Symptom Index and Nepean Dyspepsia Life Quality Index in patients with FD (44). Therefore, gender can also be regarded as an underlying factor in guiding the individualized treatment of FD.

For patients with FD having MDs, psychotherapy FM combined with rabeprazole showed the best therapeutic effect. Aside from reducing GI symptoms, it improved depression at the early stages of treatment in our cohort, especially moderate depression. However, FM combined with nizatidine and FM without any AST did not improve depression early in treatment. This result is speculated to be attributed to the ability of PPIs to reduce the number of eosinophils and mast cells in the duodenum (45, 46), ultimately helping improve anxiety and depression (47, 48). Although to the best of our knowledge, no previous studies have reported the effect of FM combined with rabeprazole on patients with FD, similar treatment combinations have shown ideal effects in patients with other diseases. For example, adding amitriptyline to a PPI was more effective than a double dose of PPI in patients with functional chest pain refractory to a conventional dose of PPI (70.6 vs. 26.3%, *P* = 0.008) (49).

Additionally, when analyzing the efficacy of each treatment regimen for patients with different subtypes of FD, FM combined with rabeprazole or rabeprazole was found to improve the gastrointestinal symptoms of patients with overlapping subtypes at week 2 of treatment. PPI is by far the most effective drug for inhibiting gastric acid and has a good effect on diseases such as gastroesophageal reflux disease (50, 51), gastric ulcer bleeding (52, 53) and patients with the EPS type (13). Studies have also shown that PPI monotherapy can notably relieve dysmotility-like symptoms in patients with FD compared with H2RA plus prokinetics (54). This might be explained by the relationship between acid and gastrointestinal motility. Studies have confirmed that injecting 0.1 N hydrochloric acid (HCl) into the duodenum increases stomach sensitivity and reduces gastrointestinal motility (55). Similarly, Miwa et al. (56) demonstrated that the injection of 150 mL of 0.1 mol/L HCl into the stomach produced dyskinesia-like symptoms. However, this effect was not seen in the patients with PDS or EPS type, which could be attributed to the lower proportion of these two types of patients in this cohort compared to those previously reported (35, 57). Therefore, the relationships between PPIs and the therapeutic effect on patients with different FD subtypes require further study.

This study has limitations. First, the short follow-up (8 weeks) limited the assessment of the long-term efficacy of targeted therapy. Second, adjunctive medications (probiotics and digestive enzymes) were used in each treatment group; however, it is difficult for this study to provide a specific contribution of adjunctive medications to the improvement of GI symptoms and MDs in patients with FD, which may affect our understanding of the therapeutic effects of main medications on FD. Third, the limited sample size of each FD subtype could lead to biases in the results. Last, the cost of targeted treatment and its impact on the quality of life of patients was not determined.

Our results indicate that PPIs should be regarded as a priority treatment for patients with FD regardless of the subtype of FD or the presence of MDs compared with H2RAs. Furthermore, additional randomized controlled trials are needed to determine the mode of action of PPIs and the etiology of FD.

## Conclusion

This study identified the risk factors related to MDs in patients with FD (high GI symptom score and low educational level), provided more effective targeted treatment options for patients with and without MDs and confirmed sex differences in FM treatment. Thus, this study aids in the individualized clinical management of FD.

## Data availability statement

The raw data supporting the conclusions of this article will be made available by the authors, without undue reservation.

## Ethics statement

The studies involving human participants were reviewed and approved by the Ethics Committee of The Third Xiangya Hospital of Central South University. The patients/participants provided their written informed consent to participate in this study.

## Author contributions

FW was involved in the study design, the intervention of the research plan, and critical revision of the manuscript. QH completed follow-up of patients and collated and wrote the manuscript. SZhe participated in the analysis, interpretation of the data, and revision of the manuscript. TC, SZha, and QS participated in the registration of patient information. All authors approved the final version of the manuscript, including the authorship list.

## Funding

This research was funded by the following grants: The New Xiangya Talent Project of the Third Xiangya Hospital of Central South University (20180304), Hunan Provincial Natural Science Foundation of China (2020JJ4853), Hunan Provincial Clinical Medical Technology Innovation Guidance Project (2020SK53616), Scientific Research Project of Hunan Provincial Health Commission (202103032097), the Independent Exploration and Innovation Project of Central South University (2020zzts295), Hunan Provincial Natural Science Foundation of China for Youths (2020JJ5609).

## Conflict of interest

The authors declare that the research was conducted in the absence of any commercial or financial relationships that could be construed as a potential conflict of interest.

## Publisher's note

All claims expressed in this article are solely those of the authors and do not necessarily represent those of their affiliated organizations, or those of the publisher, the editors and the reviewers. Any product that may be evaluated in this article, or claim that may be made by its manufacturer, is not guaranteed or endorsed by the publisher.
