# Supplementary material for: Factors associated with mood disorders and the efficacy of the targeted treatment of functional dyspepsia: A randomized clinical trial
Source: Front Med (Lausanne). 2022 Jul 22;9:859661. doi: 10.3389/fmed.2022.859661 (PMC9353185; doi:10.3389/fmed.2022.859661)
Supplement: Supplementary file 2 [file Data_Sheet_2.pdf]

Supplementary Material

1 Supplementary Tables and Figures

1.1 Supplementary Tables

1.1.1 Table S1. Questionnaire on the therapeutic efficacy of different drug regimens on functional dyspepsia

General information	Name:	Sex:	Age:	Telephone:	Marital status:
	Height (meters):	Weight (kg):	Employed: □Yes □No	Educational level	Home address:
Process	When did functional dyspepsia symptoms first appear?			Duration of symptoms:	
Previous treatment	Did you receive a diagnosis of functional dyspepsia? □ Yes □ No<br>Number of previous treatments：□<3, □>=3				
Previous medications	□Prokinetics □Antacids □Mucosal protectants □Digestive enzymes				
Comorbidities	□Cardiovascular disorders □Endocrine diseases □Neurological disorders □Others				
Gastrointestinal symptoms	Before treatment	At 2 weeks of treatment	At 4 weeks of treatment	At 8 weeks of treatment	Notes
	Score	Score	Score	Score	
Postprandial fullness					
Epigastric distention					
Early satiety					
Nausea					
Belching					
Epigastric pain					
Epigastric burning					
Acid reflux					
Chest pain					
Chest burning					
Vomiting					
Bad breath					
Gastrointestinal symptom scores	0: No symptoms				
	1: A few symptoms, no impact on daily life				
	2: Some symptoms, some impact on daily life				
	3: Several symptoms, significant impact on daily life				

<table>
<tr><td rowspan="2">Examination</td><td>Gastroscopy</td><td></td><td>^{14}C urea breath test</td><td></td></tr>
<tr><td>Abdominal ultrasound</td><td></td><td>Others</td><td></td></tr>
<tr><td>Therapeutic regimen</td><td colspan="4"></td></tr>
<tr><td rowspan="6">Adverse drug reactions</td><td colspan="4">□ Yes □ No Time of appearance: Severity：□ Mild □ Moderate □ Severe</td></tr>
<tr><td colspan="4">Description:</td></tr>
<tr><td colspan="2">Need to take measures：□ Yes □ No</td><td colspan="2">Combination therapy: Treatment duration:</td></tr>
<tr><td rowspan="3">Severity</td><td colspan="3">Mild (continuous treatment)</td></tr>
<tr><td colspan="3">Moderate (treatment withdrawal, no treatment)</td></tr>
<tr><td colspan="3">Severe (treatment withdrawal, symptomatic treatment)</td></tr>
</table>

1.1.2 Table S2A. Patient Health Questionnaire-9(PHQ-9) Scale

Outpatient No: Mobile No: Name: Date:

	In the past two weeks, how often did you experience the following conditions?	Score			
		Never	Sometimes	Usually	Always
1	Lacking enthusiasm or interest in doing things	0	1	2	3
2	Feeling low, depressed, or hopeless.	0	1	2	3
3	Difficulty falling asleep, oversleeping, or restlessness	0	1	2	3
4	Feeling tired or lacking energy	0	1	2	3
5	Having increased or decreased appetite	0	1	2	3
6	Feeling frustrated or defeated, or letting yourself or your family down	0	1	2	3
7	Having difficulty doing simple things, such as reading a newspaper or watching television	0	1	2	3
8	Feeling lethargic or restless	0	1	2	3
9	Having thoughts of dying or hurting yourself	0	1	2	3
Total score (0–27)					
Outcome / Score	No depression	Mild depression	Moderate depression	Moderate to severe depression	Severe depression
Standard score	0-4	5-9	10-14	15-19	20-27
(Please choose the answer that					

best indicates the degree of depression)					

Table S2B. Generalized Anxiety Disorder-7 Scale

Outpatient No. : Mobile No. : Name: Date:

	In the past two weeks, how often did you have these feelings:	Score			
		Never	Sometimes	Usually	Always
1	Nervousness or anxiety				
2	Difficulty controlling emotions				
3	Excessive worry				
4	Difficulty relaxing				
5	Restlessness				
6	Became annoyed easily				
7	Worried that something bad could happen to you soon				
Total score (0–21)					
Outcome / Score	No anxiety	Mild anxiety	Moderate anxiety	Moderate to severe anxiety	Severe anxiety
Standard score	0-4	5-9	10-13	14-18	19-21
(Please choose the answer that best indicates the severity of your symptoms)					

1.1.3 Table S3. Baseline characteristics in patients with functional dyspepsia with mood disorders

**Feature**	Group A (n=44)	Group B (n=44)	Group C (n=113)	*P*-value
**Sex, n(%)**				0.906
Female	27(61.36)	25(56.82)	66(58.41)	
**Age, years (median, Q1-Q3)**	43.50 (32.25-56.00)	46.50 (34.00-55.00)	45.00 (31.00-54.00)	0.784
**BMI, kg/m^2 (mean ± SEM)**	21.67±3.78	22.26±2.62	21.47±2.72	0.284
**GI symptom score(median, Q1-Q3)**	9.00(7.25-11.75)	7.00(5.00-10.75)	8.00(5.00-11.00)	0.071
**Depression score(median, Q1-Q3)**	9.00(6.00-10.75)	7.00(5.00-9.00)	7.00(5.00-9.50)	0.062
**Anxiety score (median, Q1-Q3)**	7.00(5.00-12.00)	5.00(4.00-8.00)	6.00(4.00-9.00)	0.098
**Marital status, n(%)**				0.590
Others	8(18.18)	5(11.36)	20(17.70)	
Married	36(81.82)	39(88.64)	93(82.30)	
**Duration of disease onset, n(%)**				0.132
0.5-<1	8(18.18)	16(36.36)	37(32.74)	
1-<5	19(43.18)	17(38.64)	54(47.80)	
5-<10	7(15.91)	7(15.91)	11(9.73)	
≥10	10(22.73)	4(9.09)	11(9.73)	
**Number of previous treatments, n(%)**				0.683
<3	20(45.45)	24(54.55)	58(51.33)	
≥3	24(54.55)	20(45.45)	55(48.67)	
**Educational level , n(%)**				0.707
Primary	6(13.64)	6(13.64)	13(11.50)	
Secondary	21(47.72)	17(38.64)	58(51.33)	
Higher	17(38.64)	21(47.72)	42(37.17)	
**Employed, n(%)**				0.505
No	15(34.09)	17(38.64)	33(29.20)	
Yes	29(65.91)	27(61.36)	80(70.80)	
**FD subtype, n(%)**				0.462
PDS	6(13.64)	8(18.18)	29(25.66)	
EPS	6(13.64)	8(18.18)	16(14.16)	
Overlapping subtype†	32(72.72)	28(63.64)	68(60.28)	
**Cardinal symptom, (median, Q1-Q3)**				
Postprandial fullness	2.00(1.00-3.00)	1.00(0.00-2.00)	1.00(1.00-2.00)	0.160

Early satiety	0.00(0.00-2.00)	0.00(0.00-1.00)	0.00(0.00-2.00)	0.473
Epigastric pain	1.00(0.00-2.00)	1.00(0.00-1.00)	1.00(0.00-2.00)	0.644
Epigastric burning	1.00(0.00-1.00)	0.00(0.00-1.00)	0.00(0.00-1.00)	0.128
Epigastric distention	1.00(0.00-2.00)	1.00(0.00-2.00)	1.00(0.00-2.00)	0.351
Nausea	0.50(0.00-1.00)	0.00(0.00-1.00)	0.00(0.00-1.00)	0.090
Belching	1.00(0.00-2.00)	0.50(0.00-1.00)	1.00(0.00-2.00)	0.367
Acid reflux	1.00(1.00-2.00)	1.00(1.00-1.75)	-	0.199

Group A: flupentixol and melitracen + nizatidine; Group B: flupentixol and melitracen + rabeprazole; Group C: flupentixol and melitracen. Q, quartile; SEM, standard error of mean; BMI, body mass index; GI, gastrointestinal; PDS, postprandial distress syndrome; EPS, epigastric pain syndrome. †One or two symptoms of both PDS and EPS.

1.1.4 Table S4. Comparison of total remission rate of mood disorder in patients with functional dyspepsia at 2, 4, and 8 weeks of treatment (n [%]).

Treatment duration (weeks)	Group A (n=44)	Group B (n=44)	Group C (n=113)
2	20(45.45)	30(68.18)	54(47.79)
4	38(86.36)***	41(93.18)**	92(81.42)***
8	42(95.45)***#	43(97.73)**	107(94.69)***##

Group A: flupentixol and melitracen + nizatidine; Group B: flupentixol and melitracen + rabeprazole; Group C: flupentixol and melitracen. **$P < 0.01$, ***$P < 0.001$: vs. 2 weeks of treatment in the same group. #$P < 0.05$, ##$P < 0.01$: vs. 4 weeks of treatment in the same group.

1.1.5 Table S5A. Gender differences in the improvement of mood disorders in patients with FD in each treatment group.

Treatment duration (weeks)	Group A[1]	Group A[2]	*P*	Group B[1]	Group B[2]	*P*	Group C[1]	Group C[2]	*P*
2	29.41 (5/17)	55.56 (15/27)	0.09	63.16 (12/19)	72.00 (18/25)	0.53	40.43 (19/47)	53.03 (35/66)	0.19
4	94.12 (16/17)	81.48 (22/27)	0.38	94.74 (18/19)	92.00 (23/25)	1.00	76.60 (36/47)	84.85 (56/66)	0.27
8	100.00 (17/17)	92.59 (25/27)	0.52	100.00 (19/19)	96.00 (24/25)	1.00	91.49 (43/47)	96.97 (64/66)	0.23

Group A: flupentixol and melitracen + nizatidine; Group B: flupentixol and melitracen + rabeprazole; Group C: flupentixol and melitracen. [1]Remission rates of overall gastrointestinal symptoms in male patients with FD; [2]Remission rates of overall gastrointestinal symptoms in female patients with FD. Remission rate (%).

Table S5B. Gender differences in the improvement of depression in patients with FD in each treatment group.

Treatment duration (weeks)	Group A[1]	Group A[2]	*P*	Group B[1]	Group B[2]	*P*	Group C[1]	Group C[2]	*P*
2	25.00 (4/16)	55.00 (11/20)	0.07	70.59 (12/17)	73.68 (14/19)	1.00	27.50 (11/40)	53.06 (26/49)	0.015
4	87.50 (14/16)	85.00 (17/20)	1.00	94.12 (16/17)	84.21 (16/19)	0.61	80.00 (32/40)	81.63 (40/49)	0.85
8	100.00 (16/16)	90.00 (18/20)	0.50	100.00 (17/17)	94.74 (18/19)	1.00	87.50 (35/40)	95.92 (47/49)	0.24

Group A: flupentixol and melitracen + nizatidine; Group B: flupentixol and melitracen + rabeprazole; Group C: flupentixol and melitracen. [1]Remission rates of overall gastrointestinal symptoms in male patients with FD; [2]Remission rates of overall gastrointestinal symptoms in female patients with FD. Remission rate (%).

Table S5C. Gender differences in the improvement of anxiety in patients with FD in each treatment group.

Treatment duration (weeks)	Group A[1]	Group A[2]	*P*	Group B[1]	Group B[2]	*P*	Group C[1]	Group C[2]	*P*
2	54.55 (6/11)	57.69 (15/26)	1.00	53.85 (7/13)	73.68 (14/19)	0.28	43.75 (14/32)	58.33 (28/48)	0.20
4	81.82 (9/11)	80.77 (21/26)	1.00	84.62 (11/13)	94.74 (18/19)	0.55	75.00 (24/32)	85.42 (41/48)	0.24
8	100.00 (11/11)	92.31 (24/26)	0.57	100.00 (13/13)	94.74 (18/19)	1.00	93.75 (30/32)	93.75 (45/48)	1.00

Group A: flupentixol and melitracen + nizatidine; Group B: flupentixol and melitracen + rabeprazole; Group C: flupentixol and melitracen. [1]Remission rates of overall gastrointestinal symptoms in male patients with FD; [2]Remission rates of overall gastrointestinal symptoms in female patients with FD. Remission rate (%).

1.1.6 Table S6. Baseline characteristics in patients with functional dyspepsia without mood disorders

**Feature**	Group D (n=68)	Group E (n=68)	*P*-value
**Sex, n(%)**			0.607
Female	33(48.53)	36(52.94)	
**Age, years (mean ± SEM)**	43.74±13.05	46.51±12.64	0.209
**BMI, kg/m² (median, Q1-Q3)**	22.46(19.82-24.51)	22.45(20.07-24.52)	0.729
**GI symptom score(median, Q1-Q3)**	6.00(4.00-9.75)	5.00(4.00-8.00)	0.104
**Marital status, n(%)**			0.055
Others	11(16.18)	4(5.88)	
Married	57(83.82)	64(94.12)	
**Duration of disease onset, n(%)**			0.148
0.5-<1	21(30.88)	28(41.18)	
1-<5	29(42.65)	20(29.41)	
5-<10	7(10.29)	13(19.12)	
≥10	11(16.18)	7(10.29)	
**Number of previous treatments, n(%)**			0.059
<3	31(45.59)	42(61.76)	
≥3	37(54.41)	26(38.24)	
**Educational level , n(%)**			0.615
Primary	4(5.88)	2(2.94)	
Secondary	30(44.12)	34(50.00)	
Higher	34(50.00)	32(47.06)	
**Employed, n(%)**			0.244
No	15(22.09)	21(30.88)	
Yes	53(77.91)	47(69.12)	
**FD subtype, n(%)**			0.874
PDS	18(26.47)	20(29.41)	
EPS	14(20.59)	15(22.09)	
Overlapping subtype†	36(52.94)	33(48.50)	
**Cardinal symptom, (median, Q1-Q3)**			
Postprandial fullness	1.00(0.00-2.00)	1.00(0.00-2.00)	0.481
Early satiety	0.00(0.00-1.00)	0.00(0.00-0.75)	0.058
Epigastric pain	1.00(0.00-2.00)	1.00(0.00-1.00)	0.079
Epigastric burning	0.00(0.00-0.00)	0.00(0.00-0.00)	0.874
Epigastric distention	0.50(0.00-2.00)	1.00(0.00-1.75)	0.675
Nausea	0.00(0.00-1.00)	0.00(0.00-1.00)	0.911
Belching	0.00(0.00-2.00)	1.00(0.00-1.00)	0.821
Acid reflux	0.00(0.00-1.00)	0.00(0.00-1.00)	0.204

Group D: nizatidine; Group E: rabeprazole. BMI, body mass index; Q, quartile; GI, gastrointestinal; PDS, postprandial distress syndrome; EPS, epigastric pain syndrome. †One or two symptoms of both PDS and EPS.

2.2 Supplementary Figures

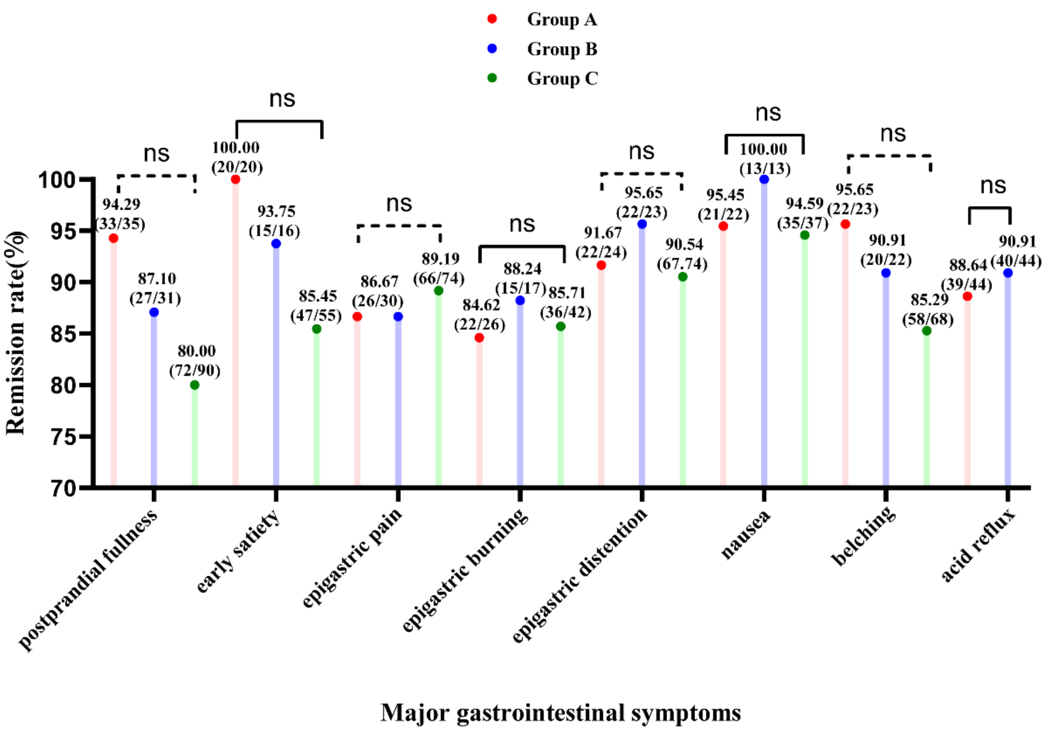

Figure S1 Comparison of relief rates of major gastrointestinal symptoms between groups A, B, and C at 8 weeks of treatment. Ns: no significance between groups A, B and C.

A

B

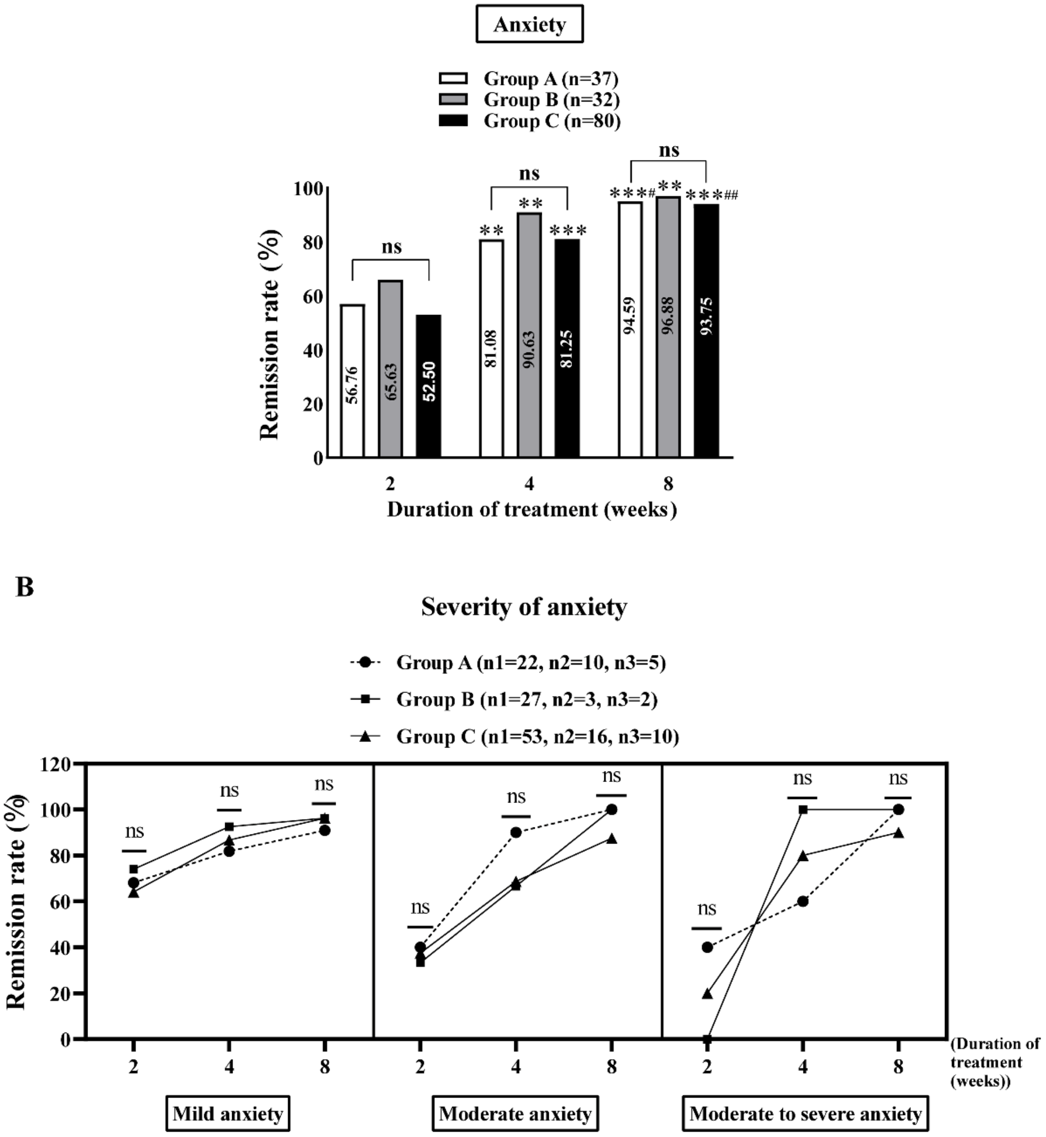

Figure S2 Improvement of anxiety in the study population. Comparison of the remission rates of general anxiety **(A)** and different degrees of anxiety **(B)** in groups A, B, and C at 2, 4, and 8 weeks of treatment. Group A: flupentixol and melitracen + nizatidine; Group B: flupentixol and melitracen + rabeprazole; Group C: flupentixol and melitracen. n1, n2, and n3 correspond to the number of patients with mild, moderate, and moderate/severe anxiety, respectively. $^{**}P < 0.01$, $^{***}P < 0.001$: vs. 2 weeks of treatment in the same group. $^{\#}P < 0.05$, $^{\#\#}P < 0.01$: vs. 4 weeks of treatment in the same group; Ns: no significance between groups A, B and C.
